# Spatial Pattern Switching Enables Cyclic Evolution in Spatial Epidemics

**DOI:** 10.1371/journal.pcbi.1001030

**Published:** 2010-12-09

**Authors:** Maarten Chris Boerlijst, Willem Marijn van Ballegooijen

**Affiliations:** Theoretical Ecology, Institute for Biodiversity and Ecosystem Dynamics, University of Amsterdam, Amsterdam, The Netherlands; University of New South Wales, Australia

## Abstract

Infectious diseases often spread as spatial epidemic outbreak waves. A number of model studies have shown that such spatial pattern formation can have important consequences for the evolution of pathogens. Here, we show that such spatial patterns can cause cyclic evolutionary dynamics in selection for the length of the infectious period. The necessary reversal in the direction of selection is enabled by a qualitative change in the spatial pattern from epidemic waves to irregular local outbreaks. The spatial patterns are an emergent property of the epidemic system, and they are robust against changes in specific model assumptions. Our results indicate that emergent spatial patterns can act as a rich source for complexity in pathogen evolution.

## Author Summary

Parasites are commonly believed to evolve to make as many infections as possible. In large scale simulations of disease spread, however, natural selection can instead act to maximize outbreak frequency. Here, pathogens that cause short infections can be rewarded for their prudence by a rapid subsequent outbreak. Very surprisingly, in a simple spatial model for disease spread so-called evolutionary cycling can be observed, that is, the infection period indefinitely keeps evolving up and down. The reversal in the direction of selection is triggered by a switch in the spatial patterns from epidemic waves to irregular local outbreaks. This finding offers an alternative mechanism to explain why infectious diseases show so much variation in the lengths of their infectious periods.

## Introduction

Recent studies show that in spatial models, evolutionary dynamics of infectious diseases change with respect to predictions from non-spatial model, which implicitly assume complete mixing of individuals [1]–[5]. Spatial epidemic waves have been observed for several diseases [6]–[9]. Starting with Rand *et al.* [10], a number of authors have analyzed a spatial pathogen-host model in which a host population with local reproduction is infected by a lethal pathogen that is transmitted through direct local contact [4], [11]–[13]. In this class of models, local extinction of host populations caused by the pathogen is balanced by re-colonization of empty space by reproduction of uninfected hosts. Evolution towards increased transmissibility is limited, because pathogens that are too infectious exhaust their local host population before enough new hosts are born for the pathogen to persist.

Spatial pattern formation can also act to limit the duration of the infectious period. We recently examined a spatial epidemic model in which infection of hosts leads to waning immunity instead of host death [14], and we found that, after the system self-organizes into epidemic waves, natural selection is directed toward increasing outbreak frequency. Outbreak frequency is optimal for infections of relatively short duration so that pathogens evolve towards short lasting infections. The mechanistic explanation for competition for frequency between waves is that, when two infection waves collide, this typically is followed by local extinction of the pathogen. Subsequently, the pathogen with the higher outbreak frequency will be the first to reinvade the susceptible host population. Frequency selection also occurs in spatial competition in chemical reactions [15], autocatalytic hypercycles [16] and parasitoid-host systems [1], [17].

Our previous analysis [14] adopted a standard evolutionary approach in that ecological dynamics were operating on a faster timescale than mutation and evolution. However, for many pathogens, in particular viruses and bacteria, this assumption need not be true, as even within a single host the pathogen can accumulate many mutants as a by-product of the large mutation rate, large copy numbers, and short generation time [18], [19]. In this paper we investigate the effects of increasing mutation rate in our spatial disease model (see Model section for a description of the model). It will turn out that co-occurrence of pathogens with a substantial difference in infection period can trigger a novel type of spatial patterns, which can temporarily reverse the selection pressure in the system. As a result, the model exhibits so-called evolutionary cycling in the length of the infection period.

## Model

In our spatial epidemic model, hosts are situated in a regular square contact network. Hosts can be in three states: susceptible (*S*), infected (*I*), or resistant to infection (*R*). There are three possible state transitions, namely infection, acquiring resistance, and loss of resistance. Infection is a local contact process, where each infected host can infect its eight direct neighbors, and this occurs at rate *β*. Acquiring resistance occurs after a fixed time *τ_i_* since infection (i.e. *τ_i_* can be interpreted as the duration of the infectious period). Loss of resistance occurs after a fixed duration *τ_r_*. Note that only infection is a spatial process. Because the total population size of hosts is constant, infection is both density and frequency dependent. All time units are relative to the resistant period (which is set at *τ_r_* = 1). In this paper, we investigate evolution of the length of the infectious period. Upon each new infection, the infectious period can, with equal probability, increase or decrease with a fixed mutation size step ±Δ*τ_i_* with mutation probability *μ*. As a consequence, strains with different infectious period will co-occur and compete for susceptible hosts. We assume full cross-resistance, and no co-infection or super-infection between strains, so that after infection by a particular strain a host cannot be infected by any other strain.

In our earlier paper [14], we used a probabilistic cellular automaton model with a small fixed time step. We tested decreasing the time step until the behavior approached a continuous time process. However, such a method requires quite small time steps, which implies a large waste of computational power, as most time is used for updating hosts that do nothing within the time step. Instead, here we adopt a much faster continuous time method by using an event based updating procedure. For each infected individual, we schedule a next infection event, using an exponential distribution with rate parameter *β*. A scheduled infection event will only cause a new infection if, at the time of the event, the individual is still infectious, and if a randomly chosen neighbor individual is susceptible to infection. Acquiring resistance and loss of resistance of individuals are also scheduled as events. All events are efficiently managed in a binary heap data structure [20]. The next scheduled event is always located at the top of the binary tree, and updating the tree is an efficient process (scaling with the logarithm of the number of events). The method is vey flexible, as e.g. other probability distributions for the infectious period, such as lognormal or gamma distributions, can be implemented without loss of computational speed. Furthermore, using larger neighborhoods for the infection process does not substantially increase the computation time, as the neighborhood is only evaluated at the time of a potential infection. A non-spatial analogue of the model can be investigated by implementing infection at a global scale, that is, an infected individual can infect any other individual in the system with equal probability, thus effectively removing the spatial component from the model. A deterministic continuous approximation of the mean field dynamics [21] would consist of a set of three (SIR type) delay differential equations. Typical single run simulations of the spatial model, as reported in this paper, take around one hour to run on a Pentium computer. For large simulations several nodes of the Dutch national computer cluster LISA were used overnight. A C-code for the updating procedure can be obtained from the corresponding author upon request.

## Results

### Rock-Scissor-Paper Dynamics

**Figure 1:**
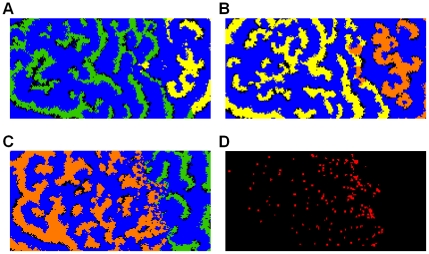
Rock-scissor-paper cyclic dominance. **A:** After 50 time units, infection period *τ_I_* = 0.4 (green) is winning against *τ_I_* = 0.6 (yellow); black indicates susceptible individuals and blue is resistance. **B:** After 50 time units, *τ_I_* = 0.6 (yellow) is winning against *τ_I_* = 0.8 (orange). **C:** After 250 time units, *τ_I_* = 0.8 (orange) is winning against *τ_I_* = 0.4 (green). **D:** Local extinction of pathogens in Fig. 1C. Here, cells are colored red if they persistently have been in direct contact with the disease during the last 10 time units; black cells indicate local extinction. All simulations are on a 201×100 field size with empty boundary conditions. Infectivity is set at *β* = 32, and resistant period *τ_R_* = 1. For initial conditions a susceptible field is seeded with 100 randomly located 10×10 blocks of infected individuals and subsequently with 100 random (overlapping) 10×10 blocks of resistant individuals. Each competitor is introduced exclusively on one side of the field, and the middle vertical row is kept susceptible for the first 10 time units, in order to let the spatial patterns develop before the competition is started. For movies see supporting Videos S1, S2 and S3.

In Fig. 1A through 1C (for movies see supplemental Videos S1, S2, S3), we investigate pair-wise competition between three pathogen genotypes that differ in infection period, with infection periods of *τ_I_* = 0.4, *τ_I_* = 0.6 and *τ_I_* = 0.8, respectively. We observe that these three pathogen types have a cyclical dominance structure, where each infection period can outcompete exactly one other infection period, resulting in a so-called “rock-scissor-paper” dynamics. In each pair-wise competition two distinct genotypes are initialized each on one side of a 201×100 field. Infection and resistance is randomly initialized in large blocks, in order to speed up the pattern formation. The middle vertical row is kept susceptible for the first 10 time units, so that the patterns can fully develop before the competition is started. Fig. 1A, *τ_I_* = 0.4 against *τ_I_* = 0.6, and Fig. 1B, *τ_I_* = 0.6 against *τ_I_* = 0.8, both show that the genotype with the shorter infection period wins the competition. This outcome is surprising, as a shorter infection period is a disadvantage at an individual level, as it generates fewer secondary infections. This selection for shorter infection period was also reported in our previous paper [14], and it can be explained by selection for higher outbreak frequency. At the spatial location where the two genotypes co-occur, the disease locally goes extinct after each wave of infection. The genotype that subsequently can cause the next disease outbreak faster will reinvade the area more quickly and it will gradually increase its domain of dominance. This selection for shorter infection period depends on the spatial pattern formation, and particularly on the epidemic waves that cause local extinction of the disease. In the non-spatial analogue of our model a shorter infection period is always a competitive disadvantage, as it locally generates less secondary infections per infected individual (i.e. it has a reduced reproduction number *R_0_*; see ref. [22]). The spatial patterns cancel this selection at the local individual level, because after each epidemic wave both disease genotypes locally will go extinct.

However, in Fig. 1C, for a two-fold difference in infection period of *τ_I_* = 0.4 against *τ_I_* = 0.8, the genotype with the longer infection period wins. This reversal of selection is accompanied by a local change in the spatial pattern; that is, at the interface between the two genotypes the dynamics now switches to a fine-scaled irregular outbreak pattern instead of regular epidemic waves. Here, the epidemic waves from the fast genotype run into remaining resistance fragments of the slow genotype. As a consequence, the waves break up and become irregular, and, most importantly, the disease no longer locally goes extinct (Fig. 1D). In the absence of local extinction, selection will favor the disease genotype with the longer infection period, because it locally generates more secondary infections (i.e. it has a larger *R_0_*, see ref. [20]).

Summing up, we have two opposite directions of selection in our system, depending on the spatial pattern at the competition interface. When the difference between infection periods is relatively small, the spatial pattern consists of epidemic waves, and both genotypes will go extinct in between successive epidemic waves. In this spatial regime, selection is for increased outbreak frequency, because the higher frequency genotype will be able to reinvade the area more quickly. In contrast, when the difference between infection periods is large, the spatial pattern at the competition interface will be irregular (i.e. turbulent), and local extinction of both genotypes between successive epidemic waves does no longer occur. In this spatial regime, selection is for increased infection period, because the genotype with longer infection period will be able to locally cause more secondary infections. In a way, the situation is reminiscent of a life history trade-off, or in particular “r” versus “K” selection theory [23], [24]. Here, the epidemic waves create an unstable “r” strategy environment, selecting for fast regeneration and growth, whereas the local irregular outbreak pattern creates a (relatively) stable “K” strategy environment, selecting for increased local competition strength. In the next paragraph we will quantify the exact boundaries for these two modes of selection, depending on the infection periods of both competitors.

### Evolutionary Cycling

**Figure 2:**
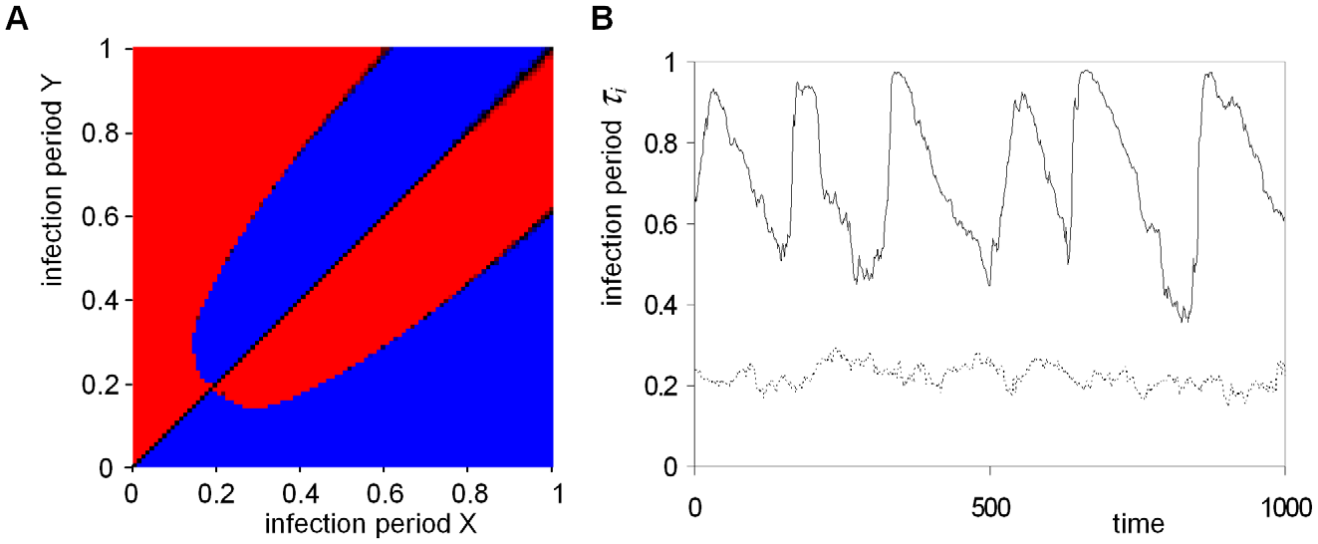
Evolutionary cycling in the length of infection period. **A:** Pair wise competition plot for infection periods. All competitions between infection periods from *τ_I_* = 0.01 up to *τ_I_* = 1 with 0.01 increment are performed using the procedure of Fig. 1 (using a 501×100 field size). Blue indicates that infection period X wins and red indicates that Y wins. Black cells indicate that the competition is not decided after 500 time units, and cells are dark shaded in blue or red if after this period there is one type that has a large majority. The figure is, as expected, almost completely symmetric along the diagonal. **B:** Attractor dynamics with small mutation rate *μ* = 0.01 (dashed line) and large mutation rate *μ* = 0.05 (solid line). Initial dynamics are omitted and only the attractor dynamics are shown for 1,000 time units. The small mutation rate converges to an attractor around *τ_I_* = 0.2, whereas the large mutation rate shows sustained large amplitude cycles in the length of the infection period. The average length of the infection period is plotted for a 40×40 subfield of a total field size of 300×300 cells with periodic boundary conditions. Mutation step size is Δ*τ_i_* = 0.01. Other parameters and settings are as in Fig. 1.

**Figure 3:**
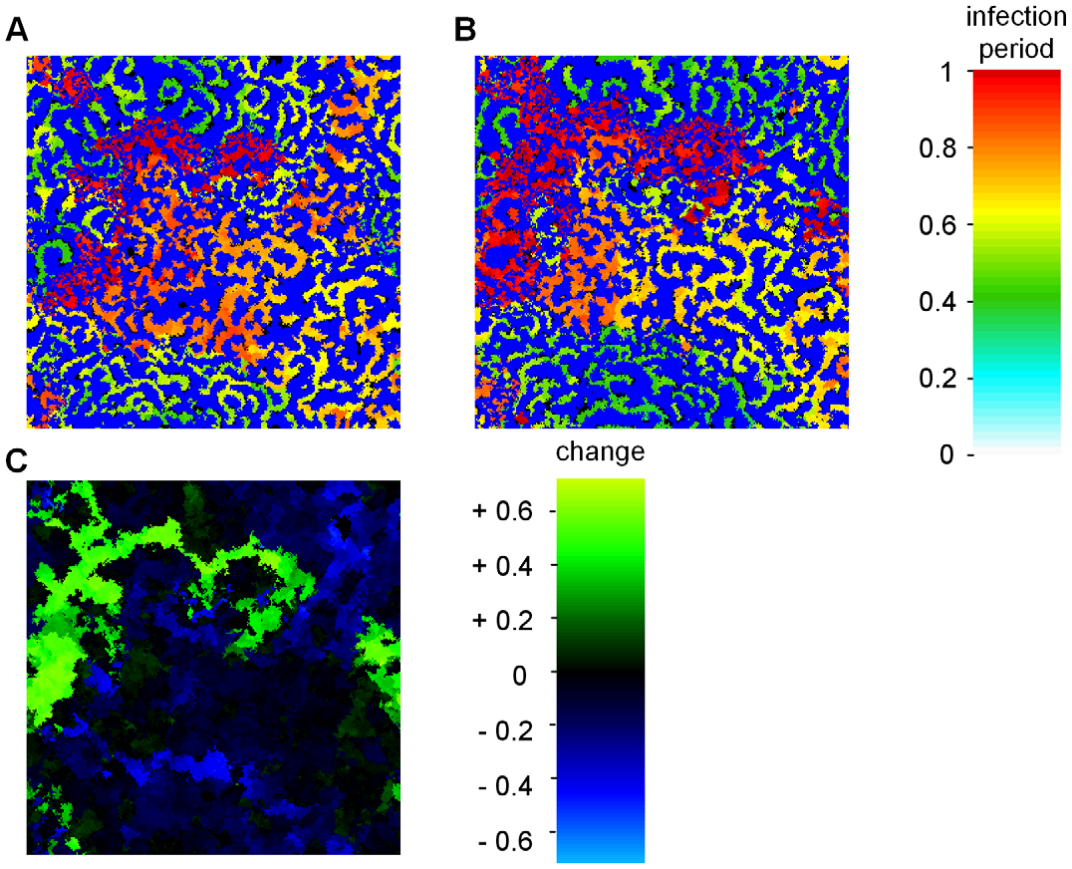
Spatial distribution in the cyclic evolutionary attractor. **A:** Spatial pattern of length of infection period after 380 time units. **B:** Spatial pattern after 400 time units; **C:** Difference in infection period between A and B. Depending on the local pattern, the infection period evolves up or down. Field size is 300×300 with periodic boundary conditions; mutation rate is *μ* = 0.05, with Δ*τ_i_* = 0.01. Other parameters and settings are as in Fig. 1. For a movie see supporting Video S4.

In Fig. 2A, a pair-wise competition plot is shown for infection periods up to *τ_I_* = 1.0. For small differences in infection period, there is selection for increased outbreak frequency, leading to an evolutionary stable attractor (ESS) around *τ_i_* = 0.2 (i.e. in the ESS, *R_0_* = *τ_I_ β* = 6.4). However, if the difference in infection period is large, the fine-scaled local irregular outbreak pattern develops, and selection favors the genotype with the longer infection period. As a consequence, for a small mutation rate of *μ* = 0.01, an evolving population will converge to the ESS, because at any time the difference between competing genotypes will be small (Fig. 2B, dotted line). However, for a larger mutation rate of *μ* = 0.05, the evolutionary dynamics are qualitatively different, and they converge to large amplitude cycling of the infection period (Fig. 2B, solid line). In the spatial pattern of the cyclic evolutionary attractor (Fig. 3A) it appears that distinct areas differ substantially in infection period. In Fig. 3B, after 20 time units, the areas of irregular outbreaks have invaded the nearby epidemic wave areas, whereas the other areas have decreased in infection period (see supplemental Video S4). In Fig. 3C the local direction of selection for this 20 time unit period is plotted, showing a strong dependence of the direction of selection on the local spatial pattern. In areas of irregular outbreaks the infection period increases whereas in areas of epidemic waves it decreases over time.

### Phase Transition and Hysteresis

**Figure 4:**
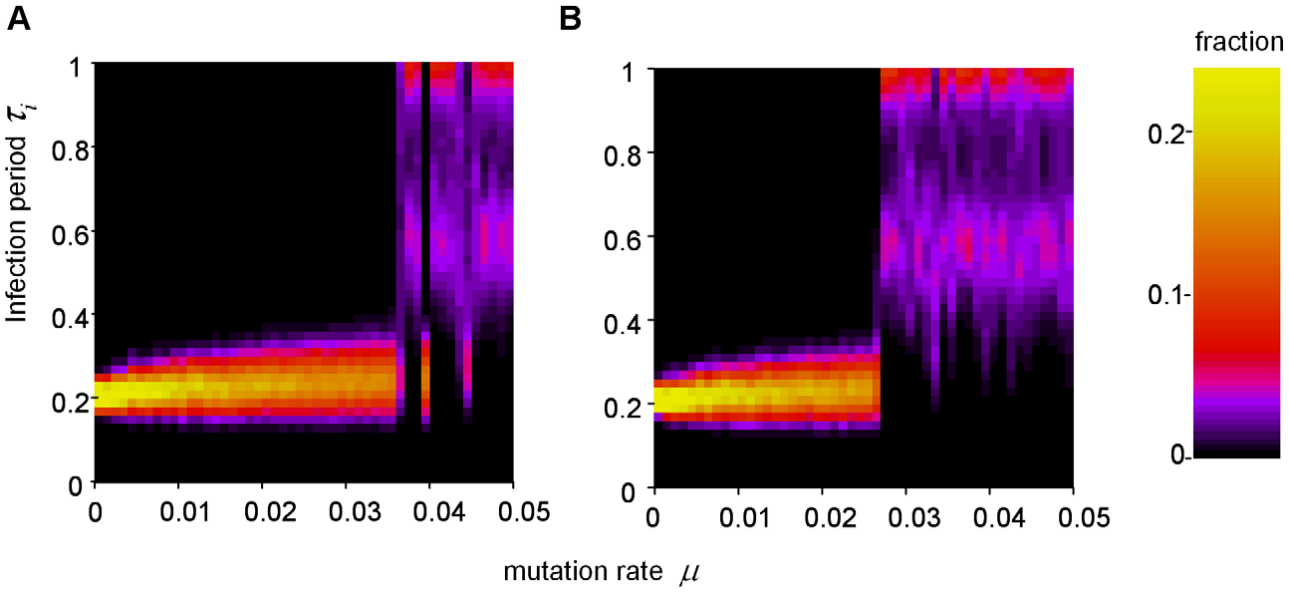
Phase transition and hysteresis in evolutionary dynamics as a function of mutation rate. **A:** Mutation rate is slowly increased starting with *μ* = 0.001 and incrementing 0.001 after each 1,000 time units. **B:** Mutation rate is slowly decreased starting with *μ* = 0.05 and decrementing 0.001 after each 1,000 time units. For each mutation rate, the average distribution of infection period is plotted for the last 200 time units. Field size is 300×300 with periodic boundary conditions. Other parameters and settings are as in Fig. 1.

When the mutation rate is slowly increased, the evolutionary dynamics show a sudden shift from the ESS to the cyclic evolutionary attractor (Fig. 4A). For increasing mutation rate, the distribution of mutants around the ESS gradually widens (due to quasispecies dynamics, see ref. [19]), until the first local irregular outbreaks can develop when neighboring genotypes differ enough in infection period. Hereafter, the dynamics quickly shifts to the cyclic attractor. If we now gradually reduce the mutation rate (Fig. 4B), it turns out that the cyclic evolutionary attractor can be maintained for quite small mutation rates, before the system falls back to the ESS. There is thus a considerable region, for 0.027≤*μ*≤0.036, where the system displays bi-stability between the ESS and the cyclic evolutionary attractor. This bi-stability can be explained by the fact that the cyclic evolutionary attractor reinforces itself, as it is associated with large differences in infection period across the field which induces the irregular outbreak pattern. It should be noted that, due to the stochastic nature of the model, the hysteresis is only a transient phenomenon; that is, allowing long enough simulation time, within the bi-stable region the dynamics will spend time in both alternative attractors. Increasing the system size will, within the bi-stable region, promote the cyclic evolutionary attractor, as the first irregular outbreaks can develop anywhere in the spatial domain, and increasing the size of the domain will thus increase the chance of the irregular outbreak pattern to develop. Once the irregular outbreak pattern has developed somewhere in the field, it will expand to the rest of the system. Also, on a small spatial domain, the cyclic evolutionary attractor is more easily lost, because persistence of the attractor depends on different spatial regions being in different phases of the attractor. On a small field global synchronization occurs more easily, and this will result in the system falling back to the ESS (i.e. *τ_i_* = 0.2) attractor.

### How Robust is the Evolutionary Cycling?

**Figure 5:**
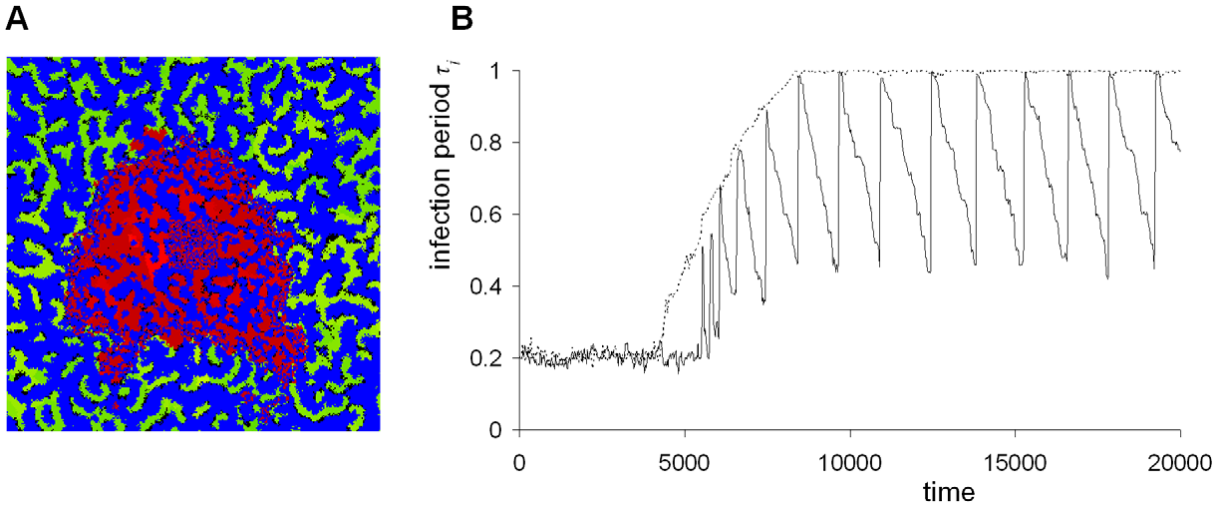
Evolutionary cycling induced by a small heterogeneous region. **A:** Pattern after 9950 time steps. The large infection periods (red) spread from a 40×40 cell heterogeneous center of the field and replace the short infection periods (green) in the periphery (for color legend see Fig. 3B). **B:** The evolutionary dynamics in the center of the field slowly evolve to maximal infection period (dotted line), and induce sustained large amplitude evolutionary cycling in the rest of the field (solid line). Field size is 300×300 cells with periodic boundary conditions. Infectivity is set at *β* = 32, and resistant period is fixed at *τ_R_* = 1. In the middle of the field, in a 40×40 cell region, the resistant period is normally distributed with mean *τ_R_* = 1 and standard deviation = 0.3. For initial conditions a susceptible field is seeded with 100 randomly located 10×10 blocks of infected individuals with *τ_I_* = 0.2 and subsequently with 100 random 10×10 blocks of resistant individuals. The average infection period is plotted for the 40×40 cells in the middle of the field (dotted line), and for 40×40 cells in the left upper corner (solid line). Mutation step size is Δ*τ_i_* = 0.01, and mutation rate is *μ* = 0.001.

In the previous section, the first local irregular outbreak pattern is generated by increasing the mutation rate. One could argue that the mutation rate that is necessary to generate this pattern is quite large, and hence the evolutionary cycling might be considered unlikely to play a role in real epidemics. However, it turns out that the irregular outbreak pattern can also originate from various other sources. For instance, cyclic evolutionary dynamics can be observed for a small mutation rate of *μ* = 0.001 (or even smaller) if only a small region of the field has some variance in the length of the resistant period. In Fig. 5 such a small inhomogeneous area is introduced in the middle of the field. Within this inhomogeneous region, the disease will develop a small scale spatial pattern in which there is no local extinction. As a consequence, within this region, the disease will evolve to maximum length of the infection period (Fig. 5, dotted line). Subsequently, the long infection periods in middle of the field will seed the irregular outbreak pattern and the resulting evolutionary cyclic dynamics in the rest of the field (Fig. 5, solid line). The mutation rate in this case will set the timescale of the evolutionary cycle, but even at very small mutation rates the evolutionary cycling dynamics persist. The evolutionary cycling here is much more regular than in Fig. 2B, because now the maximal infection period remains continuously present in the system, whereas before it occasionally was lost and had to reemerge from *de novo* mutation.

The cyclic evolutionary attractor can also be induced for small mutation rates if some local movement of individuals is included in the model, or if occasional large effect mutations are considered (results not shown). Furthermore, the occurrence of the cyclic evolutionary attractor does not depend on the specific assumptions of the model. We imposed a maximum to the length of the infection period, but such an upper limit could also be obtained implicitly by e.g. implementing a trade-off between the length of the infection period and the infectivity of a genotype. We extensively tested robustness of our results against various model assumptions, for instance by changing the contact network topology (using 4 or 6 infection neighbors), changing the boundary conditions (reflecting boundaries and empty boundary conditions), and testing various statistical distributions for the duration of the infectious period and the resistant period (e.g. log-normal and gamma distributions). The key property that is necessary for the selection for shorter infection periods to occur is recurrent local extinction of the disease. This coincides with a local dynamics that is unstable (i.e. the mean field dynamics converges to large amplitude oscillations or even extinction), inducing the spatio-temporal pattern of epidemic waves. If instead, the local (and mean field) dynamics of the disease converges to a stable endemic equilibrium, selection will favor genotypes with maximal infection period.

## Discussion

In the spatial epidemic model we present here, the local oscillating dynamics give rise to epidemic waves and recurrent local extinction of the disease. These spatial patterns enable selection for a reduced infection period, as this will increase the outbreak frequency. However, the system can switch to a local fine-scaled irregular outbreak pattern, which emerges at the interface between pathogens with large enough difference in infection period. Here, local extinction of the disease no longer occurs, and selection is in the direction of increased infection period. These opposite directions of selection can lead to large amplitude cyclic evolutionary dynamics.

Cyclic evolution was reported before for non-spatial systems [25]–[27]. It can result from co-evolution between species or traits. In contrast, in the model we present here, we observe cyclic dynamics in a single evolving trait, namely the length of the infection period. In our system, a change in the spatial pattern acts as a switch for the direction of selection. Interestingly, both alternative spatial patterns cause selection in a direction that promotes the switch to the other pattern, resulting in a continual cycling between the two patterns and directions of selection. Alternative spatial patterns can also act to induce bi-stability, in the case that each of the two patterns causes a direction of selection that enforces the current pattern [4], [17]. Note that the observed cyclic evolutionary dynamics cannot be explained with currently popular spatial analytic tools, such as spatial adaptive dynamics [28], spatial moment approximation techniques [29] or spatial inclusive fitness measurements [30]. The fine-scaled irregular outbreak pattern is only a transient pattern that occurs at the interface between genotypes, and the local selection always favors the disease genotype with longer infection period. However, as we demonstrated, this local selection can be overruled by local extinction and recolonization, which can cause selection to act in an opposite direction. In this paper, we use numerical computational methods, such as constructing the pair-wise competition plot of Fig. 2A. We feel that the current emphasis on spatial approximation techniques acts to underestimate the potential (non-linear) effects of spatial patterns on disease dynamics. In particular, spatial patterns can undergo sudden changes, such as the transition from epidemic waves to local irregular outbreaks that is observed in this paper, and such transitions are often hard (or even impossible) to predict with the current approximation techniques. Maybe these analytic tools can be improved to include, or at least predict, such transitions; for instance by developing ‘early warning signals’ for these shifts in spatial pattern [31], but this is not easy. In the meantime, we want to advocate the complementary use of numerical computational methods that incorporate the full non-linearity of the system, as such methods can increase both our qualitative and quantitative understanding of the impact of spatial pattern formation on disease dynamics.

Spatial epidemic waves have been reported for many infectious diseases [6]–[9], and recurrent local extinction is occurring for many epidemic diseases [32]. We have shown that these spatio-temporal patterns can have profound effects on selection for disease properties. Most notably, epidemic waves and recurrent local extinction can induce selection for a short infection period, which is a property of many diseases, and which is otherwise hard to explain without invoking strong trade-off assumptions [33]–[35]. We want to emphasize that the spatial patterns can induce selection for properties that emerge at a scale beyond that of the individual or the direct neighborhood of individuals [36]. Selection for increased outbreak frequency in epidemic waves is an intriguing example where a property that appears at a scale of outbreak centers overrides the local individual selection for maximizing secondary infections.

## Supporting Information

Video S1 This video shows competition between two pathogen types which differ in infection period. Infection period 0.4 (green) is winning against period 0.6 (yellow) because it causes a higher outbreak frequency. The video corresponds to Fig. 1A in the main text and has a total duration of 90 time units.
(8.70 MB AVI)

Video S2 This video shows competition between infection period 0.6 (yellow) and period 0.8 (orange). Again, the pathogen with the shorter infection period and higher outbreak frequency wins. The video corresponds to Fig. 1B and has a total duration of 100 time units.
(9.29 MB AVI)

Video S3 This video shows competition between infection period 0.8 (orange) and period 0.4 (green). Here, turbulence develops at the interface between the two types, and the type with the longer infection period slowly wins. Note that green is increasing initially, before the turbulence has developed. The video corresponds to Fig. 1C and has a total duration of 360 time units.
(10.01 MB AVI)

Video S4 This video shows cyclic evolution in the length of the infection period. The direction of selection depends on the local spatial pattern. In some areas the infection period is increasing and in other areas it is decreasing, depending on the local spatial pattern. The video corresponds to the period between Figs. 3A and 3B.
(6.00 MB AVI)
